# Prophylactic Tocilizumab prior to infusion of CD19 CAR T-cells reduces therapy-related complications in older lymphoma patients

**DOI:** 10.1007/s00277-025-06511-x

**Published:** 2025-07-25

**Authors:** Sebastian M. Stolz, Albulena Musa, Adrian Bachofner, Andriyana K. Bankova, Elise Gourri, Markus G. Manz, Max J. Rieger, Dominik Schneidawind, Nathan Wolfensberger, Thorsten Zenz, Wiebke Rösler

**Affiliations:** 1 Department of Medical Oncology and Hematology, University Hospital of Zurich, Zurich, Switzerland; 2 Medical Oncology and Hematology, Kantonsspital Winterthur, Winterthur, Switzerland; 3 Specialized Hospital for Hematological Diseases, Sofia, Bulgaria; 4 Department I of Internal Medicine, University Hospital Cologne, Cologne, Germany

**Keywords:** Tocilizumab, CAR-T, CRS, ICANS, Prophylactic, Older CAR-T patients

## Abstract

CAR (chimeric antigen receptor) T-cell therapies have transformed treatment for relapsed and refractory B cell lymphomas (r/r BCL) and plasma cell myeloma. While real-world experiences have shown success across age groups, patients over 70 years require special attention due to therapy-related complications like cytokine release syndrome (CRS) and immune effector cell-associated neurotoxicity syndrome (ICANS), which can lead to adverse outcomes. This study analyzed outcomes of 26 r/r BCL patients aged ≥ 70 years treated with CAR T-cell therapy between 2019 and 2023, comparing those who received prophylactic Tocilizumab (*N* = 7) versus standard treatment (*N* = 19). The median age was 75 years, with patients having received a median of three prior therapy lines. Prophylactic Tocilizumab was given 1 h before re-transfusion of CAR T-cells. Patients receiving prophylactic Tocilizumab experienced significantly fewer therapy-related complications (*p* = 0.039) which were defined as one or more of the following events: severe CRS, severe ICANS, corticosteroid use, need for transfusions, treatment in a critical care unit, prolonged hospitalization and discharge to a care facility. Hospital stays in total were shorter in the Tocilizumab group (15.9 vs. 18.5 days), though not statistically significant. Progression-free and overall survival were similar between groups. The results suggest that prophylactic Tocilizumab may optimize management of older CAR T-cell patients, aligning with existing evidence regarding its prophylactic use in r/r BCL.

## Introduction

Until recently, platinum-based chemotherapy, followed by high-dose therapy/autologous hematopoietic stem cell transplant (HD-CT with auto-HSCT), was considered the best option for long-term remission in relapsed/refractory diffuse-large cell B-cell lymphoma (r/r DLBCL) [1]. However, older patients were frequently considered ineligible for this intensive treatment [2]. The median age of patients with newly diagnosed DLBCL is 67 years and more than 25% of patients are older than 75 years [3, 4]. As outcomes for those who were not able to receive HD-CT and auto-HSCT following recurrence are poor, treatment for r/r DLBCL ineligible for auto-SCT and therefore mostly older patients, is a field of high medical need [5, 6]. Development of several autologous chimeric antigen receptor (CAR) T-cell products have resulted in a significant improvement in the treatment of r/r DLBCL, even more so in patients ineligible for intensive salvage therapy [7]. Alongside their therapeutical potential, treatment tolerability and outcome of CAR T-cell therapies depend on various patient- and disease-specific characteristics [8, 9]. CAR T-cells potentially cause a wide range of possible side effects such as haematological toxicities with long-lasting cytopenia, immune deficiency, therapy-related infections, cytokine-release-syndrome (CRS) and the immune effector-cell associated neurotoxicity syndrome (ICANS) [10–13]. Data regarding outcome and toxicity in older patients are heterogeneous. Several studies reported no difference regarding the outcome (CRS, ICANS, progression free survival (PFS), overall survival (OS)) of older patients compared to younger patients, while others reported a higher rate of neurological toxicities including delirium and encephalopathy [7, 14]. ^,^ [15] CRS and ICANS are usually treated with immune-modulatory drugs such as corticosteroids or Tocilizumab targeting the hyper-inflammatory state of the patient [16, 17]. CAR T-cell-related toxicities impair the treatment success, often prolong the length of hospitalization and thus cause higher health care use and cost, especially in older patients [18]. However, there is only limited data on patients aged ≥ 70 years on how these side-effects might impair the treatment outcome.

## Methods

### Data source and patient selection

We included all patients aged ≥ 70 years with r/r DLBCL, or other aggressive B-cell lymphomas treated with anti-CD19 CAR T-cell therapy in our centre from May 2019 to August 2023. The prophylactic group included patients receiving Tocilizumab 1 h prior to retransfusion of CAR T-cells as per local guidelines (age ≥ 70 years). The control group included all other patients aged ≥ 70 years treated with CAR-T cells who did not receive prophylactic tocilizumab prior to the modification of the internal guidelines.

#### Statistical analysis

Descriptive statistics were reported as median (range) for continuous variables and numbers (percentage) for categorical variables. Data were analyzed using R Studio software version 4.3.3. Missing data was dealt with by excluding patients from analyses if their file did not contain the required variables. A p-value of *p* < 0.05 was considered statistically significant. Comparisons between groups were analyzed using the Wilcoxon rank sum test (continuous data), or Fisher’s exact test (categorical data). OS and PFS was estimated using the Kaplan-Meier method, the log-rank test was used to evaluate differences between groups.

### Endpoints

The primary endpoints were PFS, OS and the G8 (geriatric screening score). Secondary endpoints included therapy-related complications such as severe ICANS (grade ≥ 3), severe CRS (grade ≥ 3), corticosteroid use, need for transfusions, treatment in a critical care unit, duration of hospitalization and discharge to a care facility.

### Ethics approval

The local ethics committee granted ethical approval for the study (BASEC No. 2023-02233) in accordance with the principles of the Declaration of Helsinki.

### Consent to participate

All patients have consented to participate via a general consent form in accordance with the specifications of the ethics application for this study.

## Results

A total of 26 patients were included in our analysis, 30.7% were female and 69.3% were male. 23 patients were diagnosed with DLBCL, 2 patients with aggressive mantle cell lymphoma and 1 patient with aggressive/transformed follicular lymphoma. Patients’ characteristics are shown in Table 1. Overall, 7 patients received prophylactic Tocilizumab infusion one hour prior to CAR T-cell retransfusion with no immediate side effects related to its administration. Only 1 patient (14.2%) received Tocilizumab again in the subsequent course due to CRS (2 additional doses) in the prophylactic group. In the control group, 7 out of 19 (36.8%) patients received Tocilizumab after CAR T-cell-retransfusion. The mean age in the Tocilizumab group was 79.1 years, compared to 73.6 years in the control group (p-value < 0.05). The median number of prior lines of therapy was three (range one to seven). Remission status was assessed according to Deauville Score and was comparable between the two groups with most patients having a progressive disease before CAR T-cell therapy. The G8 score showed a range of 12.5 to 16 points in both groups and a median of 14.1 or 14.3 respectively.

**Table 1:** Baseline characteristics

	Groups		
Characteristics	Prophylactic group N (%)	Control group N (%)	*P*– Value
Sex			
Female	1	7	
Male	6	12	
Age at Retransfusion	79.1 (R 71–85)	73.6 (R 70–78)	< 0.05
G8 Geriatric Assessment Score	14.1 (R 13.5–16)	14.3 (R 12.5–16)	ns
CAR T Product			
Lisocabtagene maraleucel	1 (14.3)	0 (0.0)	
Tisagenlecleucel	4 (57.1)	11 (57.9)	
Brexucabtagene autoleucel	0 (0.0)	2 (10.5)	
Axicabtagene ciloleucel	2 (28.6)	6 (31.6)	
Indication			
DLBCL	7 (100)	16 (84.2)	
Other NHL	0 (0.0)	3 (15.3)	
Prior Treatment Lines			ns
1	2 (28.6)	2 (10.5)	
2	3 (42.9)	4 (21.1)	
3	1 (14.3)	4 (21.1)	
4	1 (14.3)	7 (36.8)	
5	0 (0.0)	1 (5.3)	
6	0 (0.0)	0 (0.0)	
7	0 (0.0)	1 (5.3)	
Remission prior to CAR T-Cell Therapy			ns
Complete Remission	0 (0.0)	1 (5.3)	
Partial Remission	2 (28.6)	2 (10.5)	
Progressive Disease	4 (57.1)	14 (73.7)	
Stable Disease	1 (14.3)	1 (5.3)	
Not available	0 (0.0)	1 (5.3)	

**Fig. 1:**
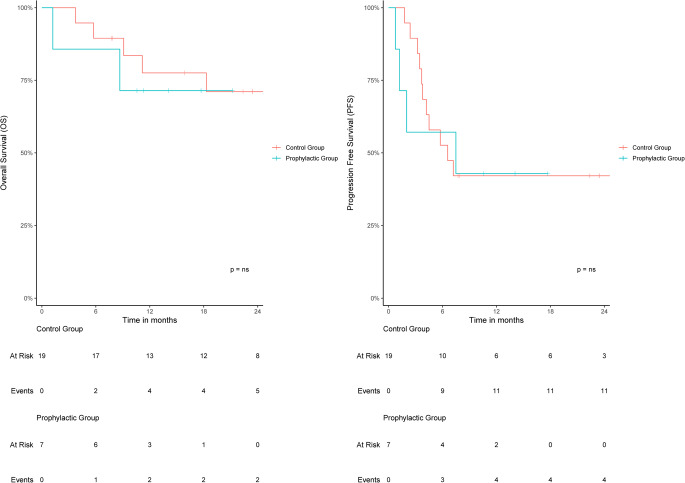
Kaplan-Meier estimates of overall survival and progression free survival were analyzed for the prophylactic tocilizumab and the control group

OS over time from CAR T-cell retransfusion is shown in Fig. 1 and did not reveal any statistically significant difference. The OS 18 months after retransfusion in the prophylactic group was 71.4% (95% confidence interval; 44.7–100%) compared to 77.5% (95% confidence interval; 60.3–99.7%) in the control group (Log-rank; p = ns). Regarding the PFS 18 months after retransfusion, there was also no statistically significant difference with a PFS of 42.9% (95% confidence interval; 18.2–100%) in the prophylactic group compared to 42.1% (24.9 − 71.3%) in the control group (p = ns). All patients without disease progression maintained in complete remission throughout the observation period, leading to the following numbers for complete remission: control-group: N:8 (42%), prophylactic group: N: 3 (42%).

**Fig. 2:**
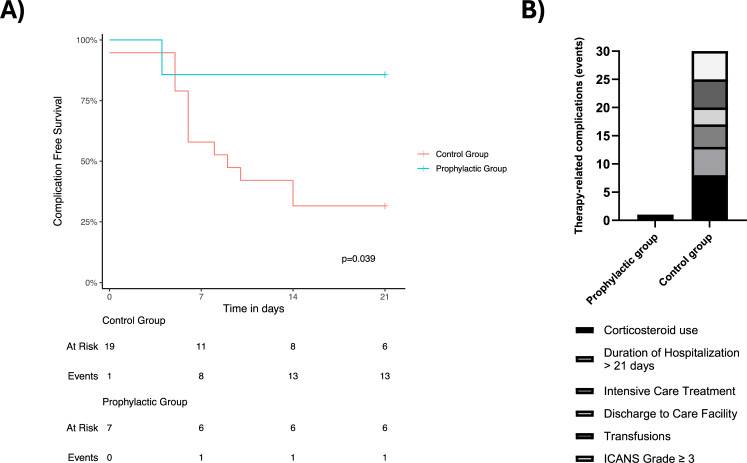
Kaplan Meier estimates of therapy-related complications (**A**) and absolute number of complications per group (**B**)

The maximum CRS grade in both groups was 2 (see Table 2). The incidence of high-grade ICANS events appeared to be lower in the prophylactic group with no grade 3 or 4 events (p = ns) compared to five patients (26.3%, 4 x Axicabtagene Ciloleucel, 1 x Brexucabtagene autoleucel) in the control group with an ICANS ≥ 3, with three patients having ICANS grade 4. Steroid use in the prophylactic group was lower but not statistically significant, where only one patient (14.3%) compared to eight patients (42.1%) received corticosteroids (p = ns). The mean duration of hospitalisation in the prophylactic group tended to be shorter with 15.9 days compared to 18.5 days in the control group (p = ns). Off note, three patients of the control group had to be discharged to a care facility (15.8%), whereas all patients in the prophylactic Tocilizumab group could be discharged home. The transfusion requirement in the control group tended to be higher (5/19) than in the prophylactic group (0/7). Using Kaplan Meier estimates, we observed a higher incidence of therapy-related complications in the control compared to the prophylactic group. This difference was statistically significant (p-value = 0.039). The distribution of these events is shown in Fig. 2.

**Table 2:** Results

	*Prophylactic group* *N* (%)	*Control group* *N* (%)	*P– Value*
CRS Events			ns
Grade 0	4 (57.1)	7 (36.8)	
Grade 1	2 (28.6)	6 (31.6)	
Grade 2	1 (14.3)	6 (31.6)	
Grade 3	0 (0)	0 (0)	
Grade 4	0 (0)	0 (0)	
ICANS Events			ns
Grade 0	4 (57.1)	10 (52.6)	
Grade 1	2 (28.6)	3 (15.8)	
Grade 2	1 (14.3)	1 (5.3)	
Grade 3	0 (0.0)	2 (10.5)	
Grade 4	0 (0.0)	3 (15.8)	
Corticosteroids			ns
Yes	1 (14.3)	8 (42.1)	
No	6 (85.7)	11 (57.9)	
Duration of Hospitalization (days)	15.9	18.5	ns
Hospitalization > 3 weeks	0 (0.0)	5 (26.3)	
Intensive Care Treatment			ns
Yes	0 (0.0)	4 (21.1)	
No	7 (100)	15 (78.9)	
Discharge to Care Facility			ns
Yes	0 (0.0)	3 (15.8)	
No	7 (100)	16 (84.2)	
Transfusions			ns
Yes	0 (0.0)	5 (26.3)	
No	7 (100)	14 (73.7)

## Discussion

In this single-center, retrospective cohort study we observed a reduction of therapy-related complications for patients aged ≥ 70 years in receiving prophylactic Tocilizumab prior to CAR T-cell therapy. Also, the prophylactic Tocilizumab group showed a lower rate of grade 2 CRS as well as severe ICANS (Grade 3/4), although not significant. We could not detect a difference between OS and PFS between both groups, but treatment outcome was generally better than those of other real world cohorts, potentially reflecting differences in patient selection for CAR T-cell therapy in the older population [18]. This bias may also be underlined by a median G8 score of 14, suggesting that primarily older patients considered to be “very fit” without geriatric impairments received CAR T-cell therapy. This showcases the difficulties of balancing the harms and benefits of cancer treatment in older patient, as also discussed by Mangan et al. [19]. The relevance of prophylactic Tocilizumab administration in the context of CAR T-cell therapy is not well established. Caimi et al. demonstrated in their non-comparative cohort study in 2021 a low incidence of CRS and ICANS, particularly also compared to the initial JULIET trial [20, 21]. However, a prospective (sub-) trial of the ZUMA-1 study recently published by Locke et al., suggests a high rate of severe neurologic events (grade ≥ 3; 42%) following prophylactic administration of Tocilizumab 48 h after CAR T-infusion [22]. Another approach involves administering Tocilizumab preemptively in cases of persistent CRS grade 1, as performed by De Philippis et al. [23]. In their cohort, none of the patients treated preemptively with Tocilizumab developed a CRS grade ≥ 3. However, all three publications were not focused on patients ≥ 70 years, nor did those trials include a comparative cohort and the time point of the prophylactic tocilizumab treatment was different. While the available data underlines the use of geriatric management, general recommendations for optimizing treatment in the older patient other than purely supportive measures are rare [24, 25]. For the time being, no guidelines of definite thresholds exists, which older patients are considered to be eligible for CAR T-cell treatment based on objective measurements on physical and organ function, cognition or frailty. This leads to a potential under-treatment as shown by Chiara et al. [18]. Future research on older CART-patients should prospectively include endpoints relevant for this subgroup as loss of independence and need for care facilities.

Therefore, our study hints evidence that the use of prophylactic Tocilizumab reduces therapy-related complications in older lymphoma patients receiving CAR T-cell therapy. However, there are several limitations of our study. First, ours was a small, single-centered, retrospective trial and as such carries potential biases. Furthermore, given the small patient numbers in our study, there was only significant evidence when looking at the combined incidence of therapy-related complications, rather than individual events.

In summary, our data indicates that the use of prophylactic Tocilizumab addresses an important need in the care of older patients treated with CAR T-cells for B-cell lymphoma. Our results are consistent with the existing, though limited evidence regarding prophylactic or preemptive use of Tocilizumab in r/r BCL as shown in Table 3 [21, 23]. Further multi-centered, prospective and randomized studies are necessary to develop tools to tailor treatment approaches for older patients with aggressive hematological malignancies, including the management of therapy-related toxicity and integrating geriatric assessments and interventions in a structured manner.

**Table 3:** Summary of preemptive and prophylactic use of Tocilizumab in r/r BCL

Administration	Products	CRS low grade	CRS high grade	ICANS low grade	ICANS high grade	ICU	Journal
Prophylactic (d0)	Liso-cel (1) Tisa-cel (4) Axi-cel (2) Total = 7	3 (43%)	0 (0%)	3 (43%)	0 (0%)	0 (0%)	Own work
Prophylactic (d0)	Tisa-cel (2) Own (18) Total = 20	10 (50%)	0 (0%)	4 (25%)	1 (5%)	1 (5%)	Caimi et al.(21)
Prophylactic (d2)	Axi-cel (38)	34 (89%)	1 (3%)	17** (45%)	16** (43%)	NA	Locke et al.(22)
Preemptive (G1 ≥ 24 h)	Tisa-cel (13) Axi-cel (8) Brexu-cel (2) Total = 23	19 (82%) *	0 (0%)	8 (35%) *grade not specified*		NA	De Philippis et al.(23)

*calculated according to manuscript

**defined as neurotoxicity

## Data Availability

All data can be made available up reasonable request to the corresponding author.
